# Sensory Over-Responsivity as an Added Dimension in ADHD

**DOI:** 10.3389/fnint.2019.00040

**Published:** 2019-09-06

**Authors:** Shelly J. Lane, Stacey Reynolds

**Affiliations:** ^1^Department of Occupational Therapy, College of Health and Human Science, Colorado State University, Fort Collins, CO, United States; ^2^Faculty of Health and Medicine, School of Health Sciences, University of Newcastle, Callaghan, NSW, Australia; ^3^Department of Occupational Therapy, Kathryn Lawrence Dragas Sensory Processing and Stress Evaluation Laboratory, Virginia Commonwealth University, Richmond, VA, United States

**Keywords:** sensory over-responsivity, ADHD, anxiety, cortisol, children

## Abstract

Years of research have added to our understanding of Attention Deficit Hyperactivity Disorder (ADHD). None-the-less there is still much that is poorly understood. There is a need for, and ongoing interest in, developing a deeper understanding of this disorder to optimally identify risk and better inform treatment. Here, we present a compilation of findings examining ADHD both behaviorally and using neurophysiologic markers. Drawing on early work of McIntosh and co-investigators, we examined response to sensory challenge in children with ADHD, measuring HPA activity and electrodermal response (EDR) secondary to sensory stressors. In addition, we have examined the relationship between these physiologic measures, and reports of behavioral sensory over-responsivity and anxiety. Findings suggest that sensory responsivity differentiates among children with ADHD and warrants consideration. We link these findings with research conducted both prior to and after our own work and emphasize that there a growing knowledge supporting a relationship between ADHD and sensory over-responsivity, but more research is needed. Given the call from the National Institute of Health to move toward a more dimensional diagnostic process for mental health concerns, and away from the more routine categorical diagnostic process, we suggest sensory over-responsivity as a dimension in the diagnostic process for children with ADHD.

## Introduction

Attention Deficit Hyperactivity Disorder (ADHD), is a neurodevelopmental disorder characterized by ongoing patterns of inattention and/or hyperactivity and impulsivity that impacts an individual’s functioning across multiple environments. Despite being the most commonly diagnosed mental disorder in children in the United States (Centers for Disease Control and Prevention, 2018), ADHD remains a heterogeneous disorder that is not fully understood (or agreed upon) within the medical and scientific communities. None-the-less, in the United States and other countries, ADHD diagnosis is based upon evaluation by a licensed medical professional using the Diagnostic and Statistical Manual, 5th edition (DSM-5, American Psychiatric Association, 2013) “yes/no” approach to symptom presentation. Three sub-types of ADHD were introduced with the publication of the DSM-IV and these presentations were retained in the 5th edition. These classifications are (1) Predominantly Inattentive, (2) Predominately Hyperactive/Impulsive, and (3) Combined Type. A key criticism to the current approach to the diagnosis of ADHD is a failure to recognize the condition as dimensional disorder that may contain different sub-classifications, comorbidity patterns, symptom trajectories, and neurobiological correlates (Epstein and Loren, 2013). As noted by Epstein and Loren (2013) the “DSM-5 continues to place everyone meeting diagnostic criteria into a single category which does not capture the dimensionality of underlying constructs” (p. 3). The current subtyping strategies of the DSM have also been called into question, due to both the “capricious” nature by which they are assigned and the lack of studies supporting an underlying neurobiological basis for the divisions (Valor and Tannock, 2010, p. 749; Epstein and Loren, 2013).

Researchers have sought to understand the complex features more fully associated with childhood ADHD and have begun to examine qualitative and quantitative variables that impacted the expression, or predicted outcomes, of ADHD. Diagnostic moderators such as comorbid anxiety disorder, symptom severity, and child intellectual level were all identified in the ADHD population as part of a large multimodal treatment study (March et al., 2000; Owens et al., 2003; Hinshaw, 2007). In addition, neurobiological mechanisms associated with arousal and stress responsivity have begun to gain attention within the ADHD literature. Response differences within the hypothalamic-pituitary-adrenal (HPA) axis that may be related to an altered threshold for detection of stressors has emerged as a potential feature of ADHD (Kariyawasam et al., 2002). Specifically, several investigators identified a blunted cortisol response to stress in ADHD, especially in those individuals with the combined subtype of the disorder or more severe behavioral comorbidities (King et al., 1998; Maldonado et al., 2009; Van West et al., 2009). However, in a smaller sample of studies investigators also found that some children with ADHD showed higher cortisol levels following stressors (Hong et al., 2003; White and Mulligan, 2005) and yet still other studies have found no differences between ADHD and control populations (Lackschewitz et al., 2008; Hirvikoski et al., 2009). Heterogeneity in these outcomes is likely due to a variety of factors including variability in diagnostic process, cortisol collection methods used, stressors (e.g., social threat, academic task), composition of the diagnostic group, and control for comorbidities. Interestingly, anxiety disorder was noted to be a potential moderating factor in cortisol release in both ADHD and non-ADHD populations (Greaves-Lord et al., 2007; Hastings et al., 2009), suggesting the particular importance of considering co-morbidities in ADHD research.

In parallel to the work described above, but in a separate body of research, investigators began to focus on the presence of atypical sensory responsivity in children with ADHD. While a variety of terms have been used in the literature to reflect atypical sensory responsivity, sensory modulation disorders (SMD) may best describe this group of behaviors as a type underlying sensory processing dysfunction. SMDs are characterized by an inability to respond to environmental stimuli in a way that matches the demands of the stimulus (Lane, 2019). Individuals characterized as having sensory over-responsiveness (SOR) experience sensations more intensely or for a longer duration than is normal, often resulting in “fight or flight” behaviors. For those with the sensory under-responsive (SUR) subtype of SMD, sensory stimuli in the environment are disregarded, or not responded to, resulting in the child seeming to lack initial awareness. This group may appear apathetic or lacking motivation (Miller et al., 2007). As a whole, the SOR characterization has been more widely studied in the literature and is the better established subtype (Reynolds and Lane, 2008).

Multiple investigators have established that children with ADHD have difficulties in sensory processing and regulating emotional responses to sensation based on parent report questionnaires (Dunn and Bennett, 2002; Kalpogianni, 2002; Yochman et al., 2004). These studies are limited by methodology; many of the questions on the SMD parent-report tools include behaviors that overlap with the diagnostic features associated with ADHD (e.g., emotional and social responses, activity levels). Studies using objective physiological measures to record responses to sensory stimuli began to emerge in the ADHD literature, but with inconsistent findings. Mangeot et al. (2001) used electrodermal reactivity, reflecting sympathetic nervous system activity, to measure responses to sensory stimuli in children with and without ADHD. They found that children with ADHD had greater sympathetic responses to sensory stimuli upon initial presentation compared to typical children. However, other researchers using similar electrodermal measures found no differences in responsivity between ADHD and non-ADHD groups (Herpertz et al., 2003). Prior to 2009, a single published study was identified investigating SOR as a moderating variable for electrophysiological measurement in children with ADHD. Using EEG measures, Parush et al. (2007) indicated that children with ADHD and a specific form of SOR, tactile over-responsivity or defensiveness, showed different central processing of somatosensory input relative to children with ADHD without tactile-over-responsivity. Parush et al. (2007) indicated that there was a need to further investigate SOR in children with ADHD, examining children with and without SOR using neurophysiological outcomes.

Importantly, while the research published in the first decade of the 21st century expanded the scientific community’s knowledge of ADHD, associated symptoms, and neurological processes, these studies were generally conducted in isolation. That is to say, anxiety in ADHD was studied separately from stress responsivity in ADHD, and separately from sensory responsivity in ADHD. Building from these studies, our work in the Kathryn Lawrence Dragas Sensory Processing and Stress Evaluation (SPASE) lab began a trajectory of work, considering the potential relationships between and among these constructs (i.e., sensation, anxiety, and stress responses), using a more dimensional approach. We will review this work, published between 2009 and 2012, in the proceeding section, followed by an update on the current literature to provide an understanding of this area to date.

The overall purpose of this review is to summarize the current state of understanding related to ADHD and sensory processing as well as to provide a direction for future research. Ultimately, we will suggest that atypical sensory processing is an important and often overlooked dimension in the diagnosis and classification of ADHD.

## ADHD, Cortisol, Electrodermal Response, and Anxiety: Findings From the Spase Lab

### Study 1 (Reynolds and Lane, 2009): SOR and Anxiety in Children With ADHD

Our initial work investigated the co-existence of SOR and anxiety in children with ADHD (*n* = 24) and without ADHD (*n* = 24) (Reynolds and Lane, 2009). SOR in this investigation was identified using a research version [version 1.4; (V1.4)] of the Sensory Over-Responsivity Inventory (SensOR; Miller, unpublished). The SensOR had been designed as one component of a larger tool, providing parent report of sensory responses within the range of sensory domains. V1.4 was the outgrowth of content validity, field testing, and item analysis completed on earlier versions. This version was shown to have high internal consistency (Cronbach’s α = 0.65 to 0.88) and strong construct validity, distinguishing between SOR and typical responsiveness within each sensory domain (*p* < 0.001 for each domain; Schoen et al., 2008). Items on this tool addressed sensory responses across tactile, auditory, visual, olfactory, gustatory, and movement-proprioceptive sensory domains. While still in development at the time of its use, this tool was considered optimal for that study because of the focus on the over-responsive type of sensory modulation dysfunction. Established sensory-processing tools at that point in time failed to clearly distinguish between over-responsiveness and under-responsiveness. Koziol and Budding (2012) had identified this shortcoming as problematic. Scores across sensory category, and the total SensOR score were compared with scores obtained from a typical sample.

The Revised Children’s Manifest Anxiety Scale (RCMAS; Reynolds and Richmond, 2005), a behavioral measure of anxiety, was used to assess anxiety in Study 1. The RCMAS is a 37-item self-report tool appropriate for use with children ages 6 to 19. This tool provides scores across three subscales, physiological anxiety, worry/oversensitive, and social concerns/concentration, and a total score. Each subscale and the total score have been shown to have moderate to high internal consistency (α = 0.69, 0.84, 0.72, and 0.89, respectively) (Muris et al., 2002). There are 28-items measuring anxiety, and an additional nine items making up a lie or social desirability score. High RCMAS scores are consistent with greater anxiety. High lie scale scores are interpreted to reflect inconsistent, inaccurate, or other problematic responding styles from the child. There is no overlap between RCMAS items and SensOR items, but some overlap exists between RCMAS items and diagnostic symptoms of ADHD (e.g., RCMAS items “It is hard for me to keep my mind on my school work” and “I wiggle in my seat a lot” share features of both inattention and increased activity that parallel ADHD diagnostic items). Higher scores on the RCMAS indicate greater levels of anxiety. Six-month test–retest reliability indicates stability in scores for all subscales (*rs* = 0.52 to 0.68, *p* ≤ 0.01; Turgeon and Chartrand, 2003). Our approach with this tool was to have parents read aloud the items to their child, and have the child circle a “yes” or “no” to reflect their response.

Our findings in this study supported the work of Parush et al. (2007) in documenting that children with ADHD could be differentiated based on their response to sensation. Parush et al. (2007) focused their study on tactile defensiveness, suggesting that their findings indicated atypical central processing of somatosensation possibly related to disruptions in central nervous system inhibitory systems. Like Parush et al. (2007), we identified two sub-groups of children with ADHD: those with ADHD and SOR (ADHDs) and those with ADHD but no SOR (ADHDt). Further, we found that children with ADHDs more frequently exhibited anxiety within a clinically significant range than did children with ADHDt (Reynolds and Lane, 2009). If atypical neural inhibitory mechanisms play a role in tactile defensiveness, as suggested, it is possible that faulty inhibitory mechanisms are at play for both the SOR and high anxiety we identified in our work. In a systematic review, Levy had suggested that the anxiety identified in some children with ADHD might be explained by faulty prefrontal cortex and hippocampal gating, or inhibition, of amygdalar activity (Levy, 2004). Our work adds support to this possibility. Interestingly, reductions in the inhibitory neurotransmitter GABA have been identified in cortical regions that include the somatosensory and motor cortices, in children with ADHD (Edden et al., 2012). It is possible that GABA plays a role in the combination of SOR and anxiety we found in children with ADHD. Unfortunately, we were not able to assess these neurobiologic possibilities. However, our identification of a relationship between SOR and anxiety in children with ADHD had not been previously identified, and we suggested that SOR should be considered as an additional dimension within the diagnosis of ADHD.

### Study 2 (Reynolds et al., 2010): Moderating Role of SOR

Subsequently we studied the same sample of children with and without ADHD (*n* = 48) based on their response to sensory challenge, measuring sensory responsivity, electrodermal response (EDR) to sensory challenge, and cortisol at baseline and following the sensory challenge (Reynolds et al., 2010). In this study we used a version of the Sensory Challenge Protocol developed by McIntosh et al. (1999), and described in detail elsewhere (McIntosh et al., 1999). As an overview, the protocol provided stimuli within auditory, visual, smell, touch, and movement domains. Eight stimuli within one sensory domain were presented in series, using variable inter-stimulus intervals (10–15 s). Following each set of eight stimuli the protocol moved to the next sensory domain. Overall the protocol required approximately 20 min to administer. To adequately compare changes in cortisol pre- and post-exposure to sensory stimuli, we collected two salivary cortisol samples, 5-min apart, before the sensory protocol began (baseline), and seven cortisol samples post-protocol, each 5 min apart. Salivary cortisol samples were collected by placing a plain (non-citric acid) cotton Salivette (Sarstedt, Newton, NC, United States) under the child’s tongue for 60 s prior to and following the sensory challenge. Children rested and watched a silent cartoon during the post-protocol period.

Our findings pointed to SOR as a moderator variable for the HPA response to sensory challenge, reflecting a pre-existing condition/status of the participant. We proposed that children with SOR were predisposed to respond differently to the sensory stimuli in this protocol. Interestingly, we found that the subgroup of children, those with ADHDs, showed typical cortisol responses, while children with ADHDt had the expected blunted cortisol response. These findings lead us to suggest that children with ADHD might be better understood by also examining sensory responsivity (Reynolds et al., 2010). A blunted cortisol response, such as we identified in the ADHDt children, had been identified by previously in children with ADHD as a whole, and within a population of children with ADHD and behavioral co-morbidities (King et al., 1998; Maldonado et al., 2009; Van West et al., 2009). However, these findings are quite inconsistent across studies (Kamradt et al., 2018). Kamradt et al. (2018) indicate that a link between the HPA axis and ADHD had been found for children with severe externalizing behaviors, and that these behaviors might be the driver of this relationship. Our findings of sensory responsivity as a distinguishing feature indicates a need to examine further this characteristic in children with ADHD. While we did not find severe externalizing behaviors, the behaviors associated with avoidance of sensation seen in some children with SOR might be interpreted as significant externalizing behavior. We further proposed that this different sensory responsiveness might influence optimal interventions; perhaps children with both ADHD and SOR would best respond to a dual approach to treatment, addressing both the characteristics of ADHD and those of SOR. This will require additional research.

### Study 3 (Lane et al., 2010): Differentiating SOR and ADHD

Considering that the relationship between anxiety, neuroendocrine, electrodermal, and behavioral characteristics of children with ADHD might support a unique dimensional perspective on ADHD, we further examined our data, to determine if we could predict group membership among 84 6–12-year-old children with ADHDs, ADHDt, and children with no diagnosis (typicals; TYP) (Lane et al., 2010). As before, we used the RCMAS (Reynolds and Richmond, 2005) to document anxiety and SOR was identified using the SensOR (Schoen et al., 2008).

The Sensory Challenge Protocol provided the sensory stressor and we examined EDR and cortisol measures to address our question. EDR reflects the sympathetic nervous system reaction to sensation, the stressor in this study. Our protocol included a 3-min baseline and ongoing EDR measurement during the sensory challenge protocol and for a 3-min recovery at the end of the sensory challenge protocol. EDR measurement used procedures we had successfully used in other studies (Reynolds et al., 2010), and followed the recommendations of Fowles et al. (1981). Variables of interest included average tonic electrodermal activity during baseline and recovery, and mean EDR magnitude within each sensory domain to examine sensory reactivity. Not surprisingly, it was necessary to log transform the data prior to analysis as raw data were positively skewed; this is typical of EDR data (Dawson et al., 1990, 2007; Boucsein, 1992). As noted earlier, cortisol was sampled twice at baseline and for seven 5-min intervals following completion of the sensory challenge protocol.

Using stepwise discriminative analysis we found that anxiety variables were the strongest predictors, although the overall model was further strengthened using EDR variables. The combination of these variables allowed us to correctly classify 85.6% of our total sample into groups that included ADHDs, ADHDt, TYP, and TYPs. We were able to cross validate this discriminative function, correctly classifying nearly 45% of all children. In interpreting these findings we suggested that SOR be considered a steady state characteristic, driving the child’s response to environmental sensation. Nigg (2006) had described the hyperactive/impulsive characteristics of ADHD as related to reactive control and low level neural responses that are stimulus driven. We had hypothesized that the sensory responsivity we see with SOR in children with ADHD may also be related to poor reactive control. Nigg et al. (2005) both related these behaviors to meso-limbic dopamine system. While we did not investigate this, it remains a possibility. Additional consideration of the role of anxiety, it is also possible that these children experience a prefrontal/hippocampal gating deficit, such as that suggested by Levy (2004). Additional research is required.

### Study 4 (Lane et al., 2012): SOR and Anxiety in ADHD – Cause or Co-existence?

Green and Ben-Sasson (2010) had suggested that over-responsiveness to environmental sensation may become associated with other contextual features such as specific objects and events that surround the sensory stressor. They further hypothesized that this association, and the individual’s interpretation of it as both unpredictable and uncontrollable, could result in general anxiety due to the development of phobias or avoidance, hypervigilance, hyperarousal (Lane et al., 2012). Green and Ben-Sasson (2010) termed this the primary SOR model. Our study utilized a combined data set of children with ADHD and children with Autism Spectrum Disorder (ASD) to achieve a large enough sample size (*n* = 131) to conduct a path analysis examining the primary SOR model. As reported above, we used the SensOR (Schoen et al., 2008) to identify SOR in children with ADHD. The Sensory Profile (SP; Dunn, 1999) was used for children with ASD. This tool is also a parent report tool that identifies the child’s response to everyday sensation. There are 125 items on the SP, grouped into sensory domains and factors. Scoring identifies sensory quadrants into which children “fit,” reflecting the interface between neural threshold and self-regulation/responsiveness. Cronbach’s alpha for quadrant groupings are moderate to strong (*r* = 0.87 to 0.93; Dunn, 2006). For this study, which focused on the construct of over- responsivity, we used only the Sensory Sensitivity and Sensation Avoiding quadrant scores of the Sensory Profile.

To examine the primary SOR hypothesis we used path analysis (Wright, 1921). Path analysis is an approach that represents general linear models as paths; latent variables are used in the analysis. This approach allowed us to examine mediation models in which the effect of one variable (the predictor) on an outcome variable depends on a third, or mediator, variable (MacKinnon, 2008). As such we investigated our hypothesis that response to sensory challenge would mediate the relationship between our predictor and outcome variables. We used scores from the SP or the SensOR Inventory, representing SOR, and baseline physiological variables reflecting tonic arousal at the start of the sensory challenge protocol (electrodermal activity and cortisol) as predictors, and mean EDR magnitude as the mediating variable. We included recovery electrodermal activity and total anxiety score as outcome variables reflecting both recovery from potentially altered arousal levels and generalized anxiety.

We substantiated a link between SOR and anxiety in our TYP group of children. While there is ongoing controversy relative to the presence of SOR in children and adults without another diagnosis, several investigative groups have identified individuals with SOR and no additional diagnosis (Kinnealey and Oliver, 1995; McIntosh et al., 1999; Reynolds and Lane, 2008; Van Hulle et al., 2012). We also substantiated a previously described relationship between SOR and anxiety in children with ADHD (Reynolds and Lane, 2009; Lane et al., 2010). Thus, initial findings offered a degree of confirmation of the primary SOR model proposed by Green and Ben-Sasson (2010), indicating that the presence of SOR may in fact be one cause of child anxiety.

Beyond these initial findings, our path analysis confirmed a relationship between SOR and anxiety in TYP children and children with ADHD. The EDR to sensory challenge fully mediated the relationship between baseline sympathetic activity and total anxiety scores. In addition, the EDR partially accounted for a relationship between skin conductance at baseline and at recovery. We did not find mediation between baseline measures (cortisol and SOR) and outcome measures (total anxiety and skin conductance at recovery). Thus, our path model indicated that the strength of the child’s response to sensory stimuli, as seen using the sensory challenge protocol, mediates the relationship between baseline measures of arousal and attention, and anxiety and sympathetic nervous system recovery. This too supports the primary SOR model proposed by Green and Ben-Sasson (2010) indicating that SOR may be an underlying cause of child anxiety (Lane et al., 2012). A recent paper by Amos et al. (2019), also examined this relationship in a group of children with ASD. These investigators substantiated the primary SOR model, indicating that SOR and stress were mediators between autistic traits in the general population and anxiety.

### Limitations

There are of course limitations to this body of work. Our sample sizes were small, with recruitment challenged by our desire to include children with ADHD but not co-morbid behavioral or psychological conditions (e.g., ASD, Oppositional Defiant Disorder). These smaller sample sizes limited our ability to distinguish between ADHD presentations (that is primarily inattentive, primarily hyperactive, or combination), as well as our ability to examine gender differences. Our sample was also self-referred, and inherent in this is potential selection bias. Our work in the SPASE lab has been globally focused on sensory processing, and our interest in sensory processing may have attracted a unique sample of children. An additional study limitation was our use of parent report measures to assess sensory responsivity. While there is support for the validity of parent report (Diamond and Squires, 1993; Greenfield et al., 2004), there are also notable problems with this manner of data collection for some types of behaviors (Ben-Sasson et al., 2009; Woodard et al., 2012). Additional assessment limitations relate to the ADHD diagnosis; we did not include tests of attention or repeat the diagnostic process for children with a prior diagnosis of ADHD. As these studies were designed to develop a greater understanding of the dimensions of ADHD, we did not have a comparison group with only SOR. In spite of the limitations, our findings lead us to suggest that SOR is a promising dimension, but that more research is needed to clarify and substantiate the relationships we have proposed between sensory processing, anxiety, and stress responsivity in ADHD.

## Research Developments in ADHD With Links to Sensory Responsivity

Research examining the constructs of sensory responsiveness, anxiety and physiological stress responsivity have continued to emerge in the ADHD literature, and results have filled in some of the gaps left by our original work. The purpose of this section is to succinctly review recent progress in the area of ADHD and sensory responsivity.

Multiple studies have been conducted replicating earlier findings that individuals with ADHD have a higher prevalence of atypical sensory responsivity compared to those without ADHD (Shimizu et al., 2014; Pfeiffer et al., 2015; Bijlenga et al., 2017; Panagiotidi et al., 2018). Shimizu et al. (2014) looked at correlations between sensory processing and modulation problems (as measured by the Brazilian version of the Sensory Profile) and behavioral symptomatology in children with and without ADHD (*n* = 37 per group). In addition to finding that children with ADHD had a greater prevalence of atypical sensory modulation and nearly all other sensory processing problems, these authors also found strong correlations between a variety of sensory processing and modulation impairments and inappropriate behavior and learning responses in children with ADHD. Although sensory-based sub-groups were not used in this study, the authors did examine differences in SP scores between ADHD subtypes (primarily inattentive, primarily sensory processing domain and ADHD-combined), but no significant differences were identified. These findings support the work of Engel-Yeger and Ziv-On (2011) who also found no significant differences between ADHD subtypes using an abbreviated version of the SP in a population of 58 boys with ADHD.

Pfeiffer et al. (2014) sought to determine if the response to specific sensory domain inputs was associated with inattention and hyperactivity/impulsivity (core ADHD symptoms) in children ages 5–10 years. These investigators used the Sensory Processing Measure-Home Form; a parent report tool that assesses response to sensation across the range of sensory domains, social participation and motor planning (Parham and Ecker, 2007). Similar to other reports, Pfeiffer and colleagues found greater sensory modulation concerns across all sensory domains in children with ADHD relative to age-matched controls. Interestingly, they identified no significant relationships between sensory modulation scores within visual, auditory, tactile, proprioceptive, and vestibular domains and inattention or hyperactivity/impulsivity. These results were concordant with Ghanizadeh (2011) who summarized the literature in this area and concluded that DSM-based ADHD subtypes are not distinct disorders with regard to atypical sensory processing.

While the majority of research investigating atypical sensory processing and ADHD has been conducted in children, Bijlenga et al. (2017) investigated the prevalence of sensory over and under responsivity in adults with and without ADHD. In this study, 116 adults completed an Adult/Adolescent Sensory Profile (Brown and Dunn, 2002) along with an ADHD self-rating scale. As expected, the ADHD group had more sensory symptoms across all areas of the Sensory Profile and those scores correlated significantly with ADHD symptom scores. Interestingly, 43% of adult females had sensory over and/or under responsivity compared to 22% of adult males, with SOR being more prevalent in females (32%).

Panagiotidi et al. (2018) also investigated the relationship between atypical sensory processing and ADHD traits in adults, using self-report. These investigators collected responses from two online questionnaires (Glasgow Sensory Questionnaire, Adult ADHD Self-Report Scale) in a general adult population (*n* = 234). Similar to what had already been found in diagnostic samples, these investigators found an association between ADHD traits and a higher frequency of sensory difficulties in all sensory systems (e.g., visual, tactile, and auditory). In this investigation they found this relationship for both over and under responsivity. A shortcoming of this study was that no statistical methods were used to assess gender differences or potential sensory-based subtypes. Panagiotidi’s work was in part based on earlier work by Overton (2008) and Panagiotidi et al. (2017). These investigators had proposed that one potential neural locus linking ADHD, attentional concerns, and sensory over-responsivity was atypical multi-sensory integration in the superior colliculus. Overton (2008) put forth a strong argument indicating that the superior colliculus was itself over-responsive to sensory input. This structure drives our response to phasic sensory changes in the environment and leads us to turn both head and eyes toward the novel stimulus. Research in both animals and humans indicates that lesions of the superior colliculus, or disconnecting it from the prefrontal cortex, produces distractibility. Panagiotidi et al. (2017) further identified that individuals with ADHD characteristics showed poor multisensory integration (auditory and visual), quite possibly in the superior colliculus, resulting in each stimulus being perceived independently, and potentially resulting in distractibility. These investigators acknowledged that the superior colliculus was only one structure among a network of cortical and subcortical structures also linked to multisensory integration and detection of stimulus synchrony and asynchrony.

Similar to the study by Panagiotidi and Colleagues, Ben-Sasson et al. (2014) used a large normative population to study ADHD and sensory symptomatology on a continuous scale. In this study, 922 children were followed from infancy to school age. Measures included the SensOR (Schoen et al., 2008) and the ADHD scale on the Child Behavior Checklist (Achenbach and Rescorla, 2001). The authors used a dimensional clustering approach to characterize children based on ADHD and SOR symptoms. Study findings supported prior findings indicating that SOR and ADHD are often seen together in a normative sample. Ben-Sasson et al. indicated that nearly half of their sample with elevated ADHD symptoms also evidenced SOR. Similarly, of the sample with SOR, 50% also evidenced symptoms of ADHD. These rates are similar to those found by Bijlenga et al. (2017) the adult ADHD population and in our previous studies with school age children (Lane et al., 2010). An intriguing new finding from this work was that ADHD and SOR identified in early childhood continued to present into school age; this relationship appears to be stable across several developmental years. While it is fairly well established that both ADHD and SOR are conditions that do not simply resolve with age (Rasmussen and Gillberg, 2000; Ben-Sasson et al., 2010; Van Hulle et al., 2015), the stability of their co-occurrence over time specifically suggests stability in the sensory dimension of ADHD.

Another interesting finding from the Ben-Sasson (2014) study was that girls with ADHD appeared to be at a unique risk for having tactile over-responsivity. Previous findings in children had been somewhat mixed, with some investigators indicating that girls with ADHD more frequently show tactile defensiveness (Goldsmith et al., 2006; Bröring et al., 2008), and others finding no difference between boys and girls (Cheung and Siu, 2009). Interestingly, Bijlenga et al. (2017) had also found that adult women with ADHD were more likely than men with ADHD to show sensory responsivity differences, and within those differences were more likely to experience SOR.

Given that there is over-lap between behaviors associated with poor sensory processing and ADHD, there has been some effort put toward understanding how these behaviors may change or manifest over time and how they might be better distinguished from one another. For instance, Yochman et al. (2013) documented that children with SMD could be distinguished from children with ADHD using an early form of the SPM, the Evaluation of Sensory Processing (Johnson-Ecker and Parham, 2000; Parham and Johnson-Ecker, 2002) and additional psychophysical tools. Somewhat in contrast to our findings, these investigators contended that response to sensation was in fact a means of separating children with ADHD from children with SMD. Looking at change over the developmental trajectory, Ben-Sasson et al. (2014) found that children with ADHD only, SOR only, and Combined ADHD +SOR all showed patterns of over- activity and inattention in early childhood (24–48 months). The SOR-only group displayed a decline in these symptoms throughout childhood while the ADHD and ADHD+SOR groups did not. This parallels evidence suggesting that adults with SMD do not display core deficits in attentional tasks (Mazor-Karsenty et al., 2015). One explanation for this trend is that infants who later present only with SOR may display difficulties with attention and activity levels early on due to constant over-arousal and hypervigilance toward sensory stimuli (Ben-Sasson et al., 2014). Another explanation, offered by Mazor-Karsenty et al. (2015), is that inattention and high activity levels may be a stable characteristic (trait) of those with ADHD, while individuals with SOR only may experience transient states of inattention or altered activity levels under specific sensory conditions.

Both ADHD and SOR have been shown to have a genetic link. For ADHD heritability is high (Faraone et al., 2005). There has been limited investigation into the heritability of SOR, but Goldsmith et al. (2006) and Van Hulle et al. (2012) indicate that mothers of children with SOR might pass on genes related to either SOR or psychopathology. These investigators reported that SOR exists independent of other childhood psychopathology, but also commonly alongside childhood psychopathology. Interestingly they found stronger correlations between SOR symptomatology and internalizing symptoms (including anxiety) than with externalizing symptoms (including characteristics of ADHD).

Brain imaging studies might also offer some insights. While they have produced some inconsistencies for children with ADHD, looking across these investigations and the few available on children with sensory processing disorders uncovers some interesting overlaps. For instance, Owen and colleagues found, in children with sensory processing disorders, decreases of fractional anisotropy in several regions, but of interest is the superior longitudinal fascicle. Individuals with both sensory processing disorder (Owen et al., 2013) and ADHD (Liston et al., 2011) show differences in this region. Examining these tracks in individuals with both sensory processing disorders and ADHD could prove to be fruitful.

Since our initial work examining links between sensory processing, anxiety, and stress responsivity, no other studies could be identified that have examined these constructs simultaneously in children or adults with ADHD. Researchers of HPA functioning in the ADHD population have continued using a variety of methods for inducing stress and producing variable results (e.g., Corominas-Roso et al., 2015; Raz and Leykin, 2015). Kamradt et al. (2018), using meta-analysis, indicated that no consistent significant association between ADHD and cortisol reactivity could be found. However, an identified limitation was a lack of differentiation among symptom presentations and co-morbidities in the selected studies. In contradiction to this finding, Corominas-Roso et al. (2015) found a blunted cortisol response following a social challenge in those individuals with the ADHD combined subtype, but not in those with the inattentive subtype. In our preliminary work we had identified a blunted cortisol response in those children with ADHD but only in the absence of concomitant SOR. Thus, there are multiple conflicting findings relative to HPA responses in adults and children with ADHD, suggesting a need for additional research both to explore DSM-based ADHD classifications and sensory-based subgrouping schemes.

Finally, the overlap between symptoms of anxiety and ADHD has been widely studied in recent years; and research suggests that approximately 25% of adults with ADHD also meet the criteria for generalized anxiety disorder (GAD) and that approximately 25% of adults with GAD also meet the criteria for ADHD (Van Ameringen et al., 2011; Piñeiro-Dieguez et al., 2016; Reimherr et al., 2017). In adult populations, higher anxiety has been associated with both the combined type ADHD presentation as well as with greater ADHD symptomatology (for review see Reimherr et al., 2017). In children, the rates of overlap may be even higher, with some studies estimating that 30–40% of clinically referred children with ADHD have internalizing disorders such as anxiety (Jensen et al., 2001; Tannock, 2009). In both the pediatric and adult populations, the combined presence of ADHD symptoms and anxiety is thought to lead to a more complicated behavioral manifestation with greater functional impairments. Differential diagnosis between ADHD and anxiety disorders is an ongoing challenge in clinical settings (Grogan et al., 2018) and some researchers have even suggested a distinct ADHD-Anxiety clinical subtype (Jensen et al., 2001; Reimherr et al., 2017). Interestingly, both adults and children have demonstrated a reduction in anxiety symptoms when taking medications traditionally prescribed for ADHD (i.e., methylphenidate, atomoxetine), suggesting a potential common neurobiology of the symptoms comprising each disorder (Snicova et al., 2016; Reimherr et al., 2017).

Our previous work established a link between SOR and anxiety (Reynolds and Lane, 2009; Lane et al., 2012) in children with ADHD, and this link has been well substantiated in other diagnostic groups of children and adults, particularly those with ASD (Bart et al., 2017; Carpenter et al., 2018; Syu and Lin, 2018). Within the ASD literature, testable models have been proposed that link SOR, anxiety, and autistic traits as well as aspects of physiological and behavioral stress (Green and Ben-Sasson, 2010; Amos et al., 2019). As noted earlier, Amos et al. (2019), found that SOR and stress mediated the relationship between autistic traits and anxiety, in that the relationship between autistic traits and anxiety became non-significant after accounting for the effects of SOR and stress. While no specific models have been proposed and tested outlining these relationships in the ADHD population, similar mediating relationships likely exist and warrant further study in the ADHD field.

## Discussion

Years of research have added greatly to our understanding of ADHD. None-the-less there is a need for, and ongoing interest in, developing a deeper understanding of this disorder, to optimally identify risk and better inform treatment. This is consistent with the intent of the Research Domain Criteria (RDoC) delineated by the National Institute of Mental Health (NIMH) which are designed to support a better understanding of mental health and ill-health using multiple dimensions (Morris and Cuthbert, 2012). Here, we have presented a compilation of findings from our laboratory, examining ADHD behaviorally and using neurophysiologic markers. Drawing on early work of McIntosh and co- investigators, we examined response to sensory challenge in children with ADHD, measuring HPA activity and EDR secondary to sensory stressors. In addition, we have examined the relationship between these physiologic measures, and reports of behavioral sensory over-responsivity and anxiety. Together our findings suggest that sensory responsivity is not ubiquitously present in children with ADHD, and they offer a different perspective on the variability of ADHD. The co-existence of atypical sensory processing and ADHD has been documented by a number of other investigators (Pfeiffer et al., 2014; Shimizu et al., 2014; Bijlenga et al., 2017; Panagiotidi et al., 2018), providing support for our findings. Given the call from the NIMH to move toward a more dimensional diagnostic process for mental health concerns, and away from the more routine categorical diagnostic process, we suggest sensory over-responsivity as a dimension in the diagnostic process.

Our collected findings are of interest. Not all children with ADHD show SOR, but when they do, the SOR influences other aspects of behavior. In addition, we have documented that the presence of SOR moderates the stress response to sensory challenge for both typical children and children with ADHD. That is when children identified as having SOR they release cortisol in response to threat (in this case, sensory challenge) regardless of the presence or absence of the ADHD diagnosis. In contrast, children with ADHDt showed a blunted cortisol response, a finding reported by at least some other investigators (King et al., 1998; Maldonado et al., 2009; Van West et al., 2009). Further, although anxiety has been widely reported to accompany ADHD (Jensen et al., 2001; Tannock, 2009), in our work we show that anxiety that is clinically significant is not seen in children without SOR, whether they have ADHD or not. Finally, we have shown that response to perceived sensory threat influences the ability of the child with SOR to restore levels of arousal and attention to baseline, suggesting that the influence of response to this threat may itself challenge the child’s ability to regulate his or her behavior.

An important aspect of the co-occurrence of ADHD and sensory responsivity comes in the form of an impact on activity and participation. The three cases presented in our early work (Reynolds and Lane, 2008) indicated that difficulties with sensory processing, particularly sensory over-responsivity, impacted participation in routine, everyday activities. The negative impact on activity and participation has been shown by several other investigators, in association with no diagnosis other than a sensory processing deficit (c.f. Bar-Shalita et al., 2008; Cosbey et al., 2010; Ismael et al., 2015; Chien et al., 2016) in children with ASD (c.f. Provost et al., 2009; Hochhauser and Engel-Yeger, 2010; Reynolds et al., 2011a; Little et al., 2015; Piller and Pfeiffer, 2016), in children with ADHD (c.f. Engel-Yeger and Ziv-On, 2011; Reynolds et al., 2011b; Yochman et al., 2013; Pfeiffer et al., 2014), and to some extent in adults (c.f. Meredith et al., 2016).

The RDoC encourages investigators to use multiple methodologies to understand defined constructs, and we have moved toward this goal with our work. The findings reported here reflect parameters most closely related to the arousal and regulatory systems domain of function in the RDoC, although anxiety and perceived threat also reflect the negative valence systems domain. As units of analysis we have used physiologic, behavioral, and informant-report tools that reflect and relate to sensory processing in children with ADHD. Based on our work we have suggested that sensory responsivity may be an important factor to consider beyond the DSM V diagnosis of ADHD presentation.

### Future Directions

There are several directions that could be taken to develop a dimensional understanding of ADHD. Two of these more closely examine anxiety and SOR in children with ADHD. The first involves more thoroughly embedding our understanding of these features of ADHD within the context of daily life. As noted above, this process has begun. Investigations have been conducted examining the impact of atypical sensory responsivity in children with ADHD on their participation in the activities of daily life. Examining the role played by anxiety has not been undertaken relative to occupation and participation, and links between anxiety, SOR, ADHD, and engagement in the occupations of childhood have yet to be addressed.

The second direction involves embedding this work within a neurodevelopmental trajectory. This has been done to some extent in children with SOR and no other diagnosis (Ben-Sasson et al., 2010), and to a much greater extent in children with SOR and ASD (c.f. Green et al., 2012; McCormick et al., 2015; Baranek et al., 2018; Williams et al., 2018). No longitudinal studies could be found examining sensory over-responsivity, or sensory processing deficits, in children with ADHD.

Overall there is greater depth and breadth in investigations of sensory processing concerns and their relationship with the diagnosis of ASD. This is to be expected as sensory responsivity differences form one component of the DSM V diagnostic criteria for this disorder. This body of literature is vast, and it is beyond the intent of this paper to summarize the work that has been done. However, it may be important to look to that literature and consider its application in understanding the dimension of atypical sensory responsivity in children with ADHD. In doing so we may gain a better understanding of specific neural systems and networks underlying both atypical sensory responsivity and ADHD. In fact, Koziol et al. (2011) have hypothesized that interactions between the neocortex (sensory processing, motor programming), basal ganglia (selection of appropriate perceptions and motor programs), and cerebellum (sensory and motor modulation) may underlie both atypical sensory responsiveness and ADHD. Imaging studies, such as those conducted by Marco et al. (2013) with children with sensory processing concerns and autism (Owen et al., 2013; Chang et al., 2014) would provide evidence of the relationship between the behavioral features and neural connectivity, to support or negate the hypotheses proposed by Koziol et al. (2011).

## Conclusion

In conclusion, it is time to take a dimensional perspective in understanding ADHD and its various manifestations. There is general agreement that it is, in fact, a dimensional disorder (Epstein and Loren, 2013), and that through considering ADHD from a dimensional perspective we will gain an understanding of the impact on function and optimize intervention. We may also gain a deeper understanding of neurobiological differences in this population. Anxiety has been proposed as one dimension to consider, and we propose that the atypical sensory modulation is another dimension that can add to our understanding of this disorder.

## Author Contributions

Both authors listed have made a substantial, direct and intellectual contribution to the work, and approved it for publication.

## Conflict of Interest Statement

The authors declare that the research was conducted in the absence of any commercial or financial relationships that could be construed as a potential conflict of interest.
